# Mathematics anxiety—where are we and where shall we go?

**DOI:** 10.1111/nyas.14770

**Published:** 2022-03-23

**Authors:** Krzysztof Cipora, Flavia H. Santos, Karin Kucian, Ann Dowker

**Affiliations:** ^1^ Centre for Mathematical Cognition Loughborough University Loughborough United Kingdom; ^2^ UCD Music and Math Cognition, School of Psychology University College Dublin Dublin Ireland; ^3^ Center for MR‐Research University Children's Hospital Zurich Zurich Switzerland; ^4^ Department of Experimental Psychology Oxford University Oxford United Kingdom

**Keywords:** mathematics anxiety, psychology, mathematics performance

## Abstract

In this paper, we discuss several largely undisputed claims about mathematics anxiety (MA) and propose where MA research should focus, including theoretical clarifications on what MA is and what constitutes its opposite pole; discussion of construct validity, specifically relations between self‐descriptive, neurophysiological, and cognitive measures; exploration of the discrepancy between state and trait MA and theoretical and practical consequences; discussion of the prevalence of MA and the need for establishing external criteria for estimating prevalence and a proposal for such criteria; exploration of the effects of MA in different groups, such as highly anxious and high math–performing individuals; classroom and policy applications of MA knowledge; the effects of MA outside educational settings; and the consequences of MA on mental health and well‐being.

## Graphical abstract

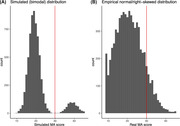

## Where are we?

Over the past seven decades,^1^ mathematics anxiety (MA) has been investigated to understand relevant psychometric, experimental, behavioral, and neurophysiological aspects. As one might expect, studies conducted over such a long period of time have brought several insights into the nature of MA. Most of these developments have been summarized in several review papers and meta‐analyses.^2^, ^3^, ^4^, ^5^, ^6^, ^7^, ^8^ We do not aim here to duplicate this vast amount of work on research synthesis but rather direct an interested reader to specific papers. We limit ourselves to relatively undisputed claims about MA (Box 1) and attempt to point to open questions and areas that, in our opinion, deserve more attention in future research.

**BOX 1. What we know about MA**

1. MA *exists*—it cannot be reduced to other constructs^3^, ^9^, ^10^
  - MA is distinct from other types of anxiety (state anxiety, trait anxiety, and test anxiety), but correlates with them positively with moderate effect sizes.^9,10^
  - MA is distinct from low mathematics performance;^5,8,9,11,12^ MA and performances are consistently found to be negatively correlated, at least in secondary pupils and adults, though usually with low to moderate effect sizes.^3,5,7–9,12^
2. MA is independent of mathematical learning disorder/developmental dyscalculia, though there are some comorbidities between the two.^13^
3. MA is present across several stages of development starting from early elementary school and its levels increase until adulthood.^5^, ^7^, ^12^
4. MA can be reliably measured with self‐descriptive instruments.^14^
5. MA is not a unidimensional construct, although there is not agreement on its dimensions. Different models assume different factor structures; the most common components refer to anxiety related to “being tested in mathematics” and anxiety related to “learning mathematics.” Another factor commonly mentioned refers to anxiety experienced in daily life situations.^14^ Some authors differentiate between cognitive, emotional, and physiological components.^14^
6. MA is linked to long‐term career choices such that individuals with elevated MA are less likely to pursue math‐intense STEM careers.^9^, ^10^
7. MA can be observed in various cultural and linguistic contexts.^3^, ^15^, ^16^ However, most studies have been conducted in developed countries.
8. Girls/women typically score higher in MA than boys/men, but this difference is usually not reflected in mathematics performance.^5^, ^9^, ^17^ This effect is present in several cultures;^18^ however, the exact reasons for the difference remain unclear.
9. Several neural (e.g., increased activation of brain regions associated with emotional response, such as amygdala or insula), physiological (e.g., skin conductance responses when solving arithmetic problems), and cognitive (e.g., reaction times and accuracies in elementary number processing tasks) correlates of MA have been identified.^4^, ^19^, ^20^
10. It is possible to alleviate MA, leading to an increase in mathematics performance. Behavioral and cognitive‐behavioral therapies have been most commonly successful.^6^, ^9^

## Blank spots and the future research agenda

### How do we understand MA?

An attentive reader might have noticed that we have yet to formally define MA. We have avoided doing so for a reason: we think that differences among definitions from different authors are not just about wording. On the contrary, they likely reflect different understandings of MA, and may, at least to some extent, contribute to *mis*understandings among researchers, and between researchers and practitioners.

One group of definitions seems to treat MA as a trait or state that can vary between individuals and between situations. For instance, Richardson and Suinn^21^ define MA as “a feeling of tension and anxiety that interferes with the manipulation of numbers and the solving of mathematical problems in (…) ordinary life and academic situations.” Similarly, Ashcraft and Ridley^10^ define it as “negative states related to mathematics and mathematical situations.”

Another group of definitions can be considered more clinical in scope. For instance, according to Lazarus,^22^ MA is “an irrational and impeditive dread of mathematics.” Tobias^23^ defines MA as “the panic, helplessness, paralysis and mental disorganization that arises among some people when they are required to solve a mathematical problem.” According to classical studies by Faust (which are cited in Ref. 10), MA meets the criteria for specific phobia. This claim is also supported by more recent work showing that behavioral and brain activation patterns of highly math anxious individuals—during exposure to mathematical problems (not even being required to solve them)—resemble patterns observed in individuals with other phobias being presented with phobia‐related stimuli.^24^ Currently, however, MA is not described in medical or psychiatric manuals and not viewed as requiring pharmacological treatment. Recently, Ashcraft postulated that MA can be understood as a personality construct, a cognitive construct, a sociocultural construct, or a neurobiological construct—which suggests that MA can be defined from several perspectives.^25^

In our view, most definitions of MA are useful; one could say that they are even very similar but differ only in degree, for example, individuals at the clinical end of the spectrum are simply those with extremely high MA. On the other hand, this might imply that only extremely high MA is detrimental to mathematics performance and/or individuals’ well‐being, or that the presence of qualitative differences distinguishes between anxious and nonanxious individuals—which, as we show in the following sections, seems not to be the case. However, it is not clearly understood at what level of anxiety MA becomes detrimental to people's performance and/or well‐being. Among other things below, we propose that when conducting future research or synthesizing existing studies, researchers should consider MA with attention to assumptions (potentially implicit) about its nature originating from different research traditions.

While there is a consensus on what characterizes high MA, there is relatively little research on the opposite pole of the MA spectrum, that is, the absence of or low MA. We offer the following potential characterizations of the pole opposite to high MA: (1) being neutral about mathematics; (2) lacking anxiety but having other negative emotions toward mathematics (e.g., anger, hate, or dislike^26^); (3) lacking anxiety but feeling resignation and helplessness; or (4) feeling positive emotions toward mathematics. Determining which of these apply and in what circumstances is not trivial. Looking at other similar constructs—for example, general anxiety—one can, on the one hand, naturally suppose that opposite to high MA along a spectrum might be the absence of anxiety. Yet, some individuals have genuine positive feeling toward mathematics (e.g., the ones who decide to pursue their careers in mathematics are passionate about it), and a few studies have looked directly at the influence of positive emotions on mathematical performance^27^, ^28^, ^29^ In contrast, some individuals experience negative emotions when encountering mathematics, even if those emotions and attitudes are not directly related to anxiety, for example, the negative emotions are dislike or boredom, or they perceive mathematics as irrelevant or useless (of course, such feelings might still co‐occur with, or even contribute to, MA in some individuals).

Being clear about definitions and exploring the entire spectrum of MA might help to integrate existing theories of MA. According to a well‐known cliché, *there is nothing more practical than a good theory*. We believe that in the case of MA this cliché is valid: better understanding what MA is, whom it affects, and where it begins/ends can guide interventions in respect of whom is targeted for therapy/help (e.g., only extremely highly anxious, (sub‐)clinical individual, or also moderately anxious, nonclinical individuals). It can also help to define the desired goal of the intervention, for example, helping participants achieve a neutral response toward mathematics or even actively liking it, or perhaps only alleviating the anxiety and not attending to (at least within the MA intervention) other negative feelings, such as boredom. It may also be that interventions should not target MA as such, but focus instead on other aspects, for example, resilience or mathematical self‐efficacy, which, while related to MA, remain distinct from it.

### Construct validity

Most studies showing construct validity of MA have focused on data from self‐description. They show that the theoretically proposed structure (e.g., components related to learning mathematics, being tested in mathematics, or encountering mathematics in everyday life (see Ref. 14 for a review)) is reflected in the factor structure observed in the data. At the same time, a recent study by Pizzie and Kraemer^30^ raises an important issue of overlap between MA and general test anxiety, especially visible in case of the MARS questionnaire.^21^ The instrument Pizzie and Kraemer developed aimed to differentiate anxiety types. While this goal was achieved, more work needs to be done to explore whether the distinction between MA and mathematics self‐concept, mathematics self‐efficacy,^16^ and mathematics attitudes remains. Importantly, differences between MA measurement instruments should be accounted for when investigating links between MA and mathematics performance.^30^ The difference between MA and statistics anxiety, a topic that has gained interest in recent years,^31^, ^32^, ^33^ is also essential to distinguish; in particular, the similarity of items measuring these two constructs necessitates further study to clarify how they are related. Among the findings are gender differences in MA and statistics anxiety, which are not as consistently found for the latter.^34^, ^35^ Additionally, the links between statistics anxiety and performance in statistics (if present at all) are weaker than those between MA and mathematics performance^32^ (but see Refs. 5 and 36).

Going beyond self‐description, studies using other measures (e.g., neurophysiological and cognitive) in the context of MA are relatively scarce and focus on group‐level differences. Studies providing converging evidence from psychometric, neurophysiological, and cognitive data remain scarce.^37^, ^38^, ^39^, ^40^, ^41^, ^42^, ^43^ Despite their relative difficulty, in comparison to questionnaires, such studies might bring new insights into the understanding of MA; they might prove particularly useful because they allow the tracing of neurophysiological/cognitive responses while performing anxiety‐inducing mathematics tasks. Nevertheless, the evidence so far shows that questionnaires remain a more reliable measure of MA than implicit MA measures.^44^ Among questions about these methods that need to be addressed are whether any correlations between self‐descriptive and neurophysiological and/or cognitive measures hold for all individuals, and whether any correlations are present when we control for other anxiety types. Looking into such questions might shed light on why, in some participants, MA (defined as scores on MA questionnaires) is linked to lower performance and avoidance of mathematics, while in others it is not.

### State trait discrepancy

The state–trait distinction is crucial in the context of general anxiety.^45^ Recent work shows that considering this distinction in the case of MA^46^, ^47^, ^48^, ^49^ might deepen theoretical understanding. It could also help with interventions, for example, it might be easier to target state anxiety than a trait characteristic. Teaching people (e.g., children/students in an educational context, but also adults who are no longer receiving formal education) strategies on how not to allow state anxiety to arise might be a useful way of alleviating the negative effects of MA. Indeed, we believe that work on state and trait aspects of MA could be fruitful for both theory building and practical implications.

### Who is math anxious: MA prevalence

**Figure 1:**
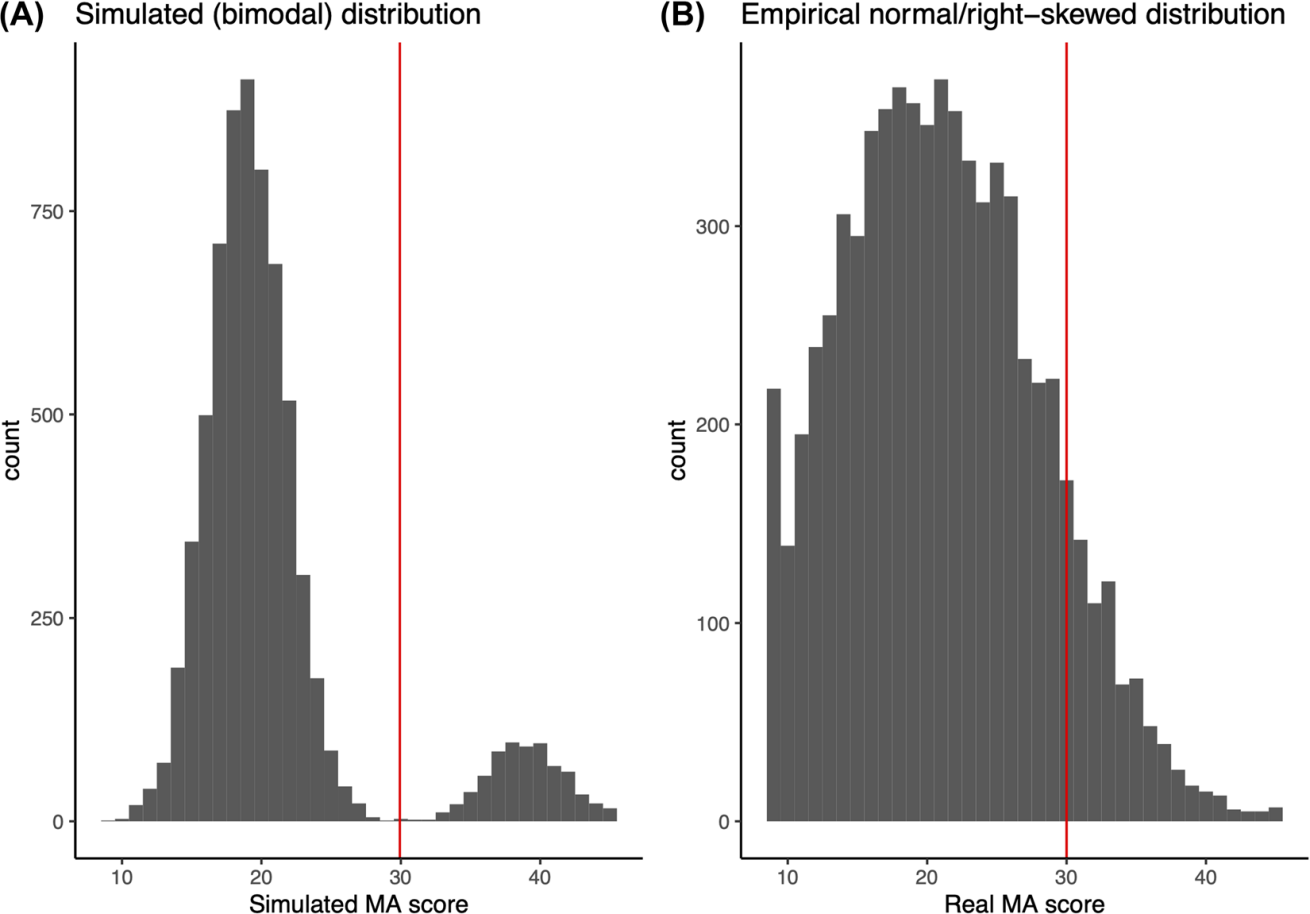
(A) Simulated bimodal distribution (7006 observations, possible range 9–45; matching the data presented in panel B). If MA scores distribution revealed two distinct groups, establishing cutoff criteria would be straightforward. (B) Empirically observed distribution of raw scores of MA questionnaire (AMAS;^52^ *n* = 7006, possible values ranging from 9 to 45 with higher values corresponding to higher anxiety) is closer to normal/right‐skewed distribution. In this case, we do not find any natural cutoff. Empirical data taken from the database by Cipora and Caviola (https://osf.io/qys6n/). In both panels, red vertical lines represent hypothetical cutoff of top 10%. The figure, AMAS data, and the R code used to generate the data are available at: https://osf.io/rjbwm/ under a CC‐BY4.0 license.

When discussing MA with nonexperts, policymakers, or educational practitioners, we often receive the reasonable question about the *prevalence* of MA. Despite being fundamental, this question still does not have a straightforward and satisfactory answer. Similar to psychological diagnostics in general, we can think of several criteria for estimating MA prevalence. Looking at distributions of different MA scale scores, we see that they either follow a normal distribution (i.e., most participants reveal scores close to sample mean and scores further away from the mean are less and less frequent), or their distribution is right‐skewed (most frequent are relatively low scores; higher scores are less and less frequent; see Fig. 1B). In any case, there are no indications that the distribution is bimodal (i.e., two relatively separate groups of scores, low and high; see Fig. 1A; see also Refs. 50 and 51).

Thus, there is no distinct, qualitative difference between anxious and nonanxious individuals—no natural differentiation between them. As is often the case in psychology, a statistical criterion can be used to classify individuals within certain percentiles on a given scale. While useful for the purpose of the individual diagnosis (e.g., an individual has MA higher than 95% of the population), it has several limitations. First, such percentile (or standard deviation–based) cutoffs are arbitrary, and there is no consensus among researchers as to which value should be used (e.g., someone in the 96th percentile might be classified as highly anxious, while individuals who fall, say, in the 94th percentile might not be classified the same way but nevertheless need support for their MA). Second, the cutoff values depend strongly on the quality and representativeness of the normative sample. The problem with the statistical criterion gets even more pronounced when one attempts to say something about the prevalence of the MA. When a statistical criterion is used (e.g., the top 10%), saying that the prevalence of MA is 10% reveals circular reasoning as it simply reiterates the adopted arbitrary statistical cutoff criterion. Another problem is the external validity of such criteria. In other words, a conclusion about whether someone is math anxious or not (aside from attempts to estimate the prevalence of MA) is solely based on the distribution of the scores in the population (approximated by means of the sample), but it does not say much about whether and how a given level of MA affects mathematical performance or well‐being. Future studies should focus on developing better diagnostic/cutoff criteria not limited to creating widely accepted and solid norms but also including external criteria for MA evaluation/diagnosis.

Use of external criteria can already be found in early studies of MA. For instance, Richardson and Suinn^21^ proposed that about 11% of a university student population has high enough MA to warrant counseling. In general, prevalence estimates available in the literature differ greatly starting from 2% to as much as 68% (see Ref. 3 for an overview). Some authors (e.g., see Ref. 54) look at how many individuals report experiencing negative emotions while doing mathematics. Such an external criterion of “feeling discomfort while doing mathematics” can be useful as a cutoff. However, one might argue that such a subjective measure should be avoided in favor of more objective ones.

We propose that the following criteria should be evaluated for MA diagnosis and intervention planning: (1) whether the MA is affecting mathematics performance beyond the current skill level (for instance, the discrepancy between math performance in relaxed, e.g., self‐paced, and stressful, e.g., time pressure, high stakes situations),^41^, ^54^, ^55^ but this method may not be useful in case of individuals characterized by very high MA levels; (2) to what extent avoidance of mathematics is a decisive factor for life/vocation choices (as in the case of adolescent/adult assessment); (3) to what extent dealing with mathematics is associated with subjective discomfort and unpleasant feelings; and (4) whether MA remits spontaneously or lasts for a (specific) period of time (similar to other forms of anxiety). We postulate that fulfilling any of the criteria 1–3, in conjunction with their persistence in time (criterion 4), would be sufficient for a diagnosis of MA.^a^ This tentative list of criteria should be supplemented by results of qualitative studies aimed at understanding potential personal effects of MA—such qualitative evaluation might become part of individual MA diagnosis. Having representative data regarding proportions of a population that meet such criteria would provide insight into both the prevalence of MA and its impact on society above and beyond looking solely at statistical criteria.

### Exploring the neglected parts of MA: performance scatterplot

As we already discussed, there is a clear link between MA and mathematics performance, with an average effect size of about −0.3, a correlation that is, at best, moderate and indicates that the common variance of MA and mathematics performance is below 10%. We believe that many studies have focused on extremes of the MA performance scatterplot, that is, individuals scoring high in MA and low in mathematics and those scoring low in MA and high in mathematics. However, what remains largely unknown are other parts of the MA versus mathematics performance scatterplot.

First, we do not know much about individuals who score around average with respect to both variables. To what extent is their mathematics performance affected by MA? Some evidence shows that MA predicts lower grades in STEM courses, even after controlling for mathematical abilities,^56^ leading us to consider the extent to which MA affects life choices and well‐being. Answering these questions would help us understand whether and to what extent addressing MA within untargeted programs aimed at general (unselected) populations is justifiable, and whether/what kind of benefits such programs could bring.

Second, there are individuals who perform well or even very well despite their (very) high math anxiety. For instance, Devine *et al*.^13^ found that around 80% of secondary schoolers scoring above the 90th percentile in MA were scoring within the typical range in mathematics (−1 SD and higher). Several studies have shown that the link between MA and mathematics performance is not uniform across different groups (a review in Ref. 14), with some studies suggesting that motivation is linked more strongly to mathematics performance than MA is,^57^, ^58^ and other studies show more complex patterns.^59^ One's self‐concept has also been shown to be significantly related to MA and mathematics performance.^28^ In addition, some STEM/mathematics students and professionals score above the minimum level in MA (e.g., see Ref. 61), yet despite having MA, these individuals successfully pursue STEM careers. It is worth understanding the factors that contribute to this. Additionally, it would be important to know (1) whether mathematics performance of these individuals remains affected (i.e., if not for MA, would they score even higher?); (2) whether MA is affecting their well‐being (and whether they might benefit from support); and (3) whether there are specific factors that help them pursue careers in STEM despite having MA (and whether knowing these factors could be used to support other highly math anxious individuals).

Conversely, we do not know much about individuals who score low in MA and yet display very low mathematics performance (e.g., some children with mathematical learning disorder/developmental dyscalculia^13^). We do not know what prevented them from developing MA within the vicious circle^2^ of MA being amplified by repeated failures in mathematics. Understanding these mechanisms can also inform the development of methods for supporting individuals with specific learning disorders, so that apart from receiving specific support in the domain of mathematics, they might also be protected from developing MA. Studying such individuals might also increase our understanding of the role of motivation: it might turn out that in some cases, both low mathematical attainment and low MA might be caused by regarding mathematics as unimportant or uninteresting, a phenomenon which needs to be distinguished from low mathematical attainment associated with dyscalculia or similar problems.

In general, we postulate that to understand MA, we should build a more thorough picture by focusing on specific groups of individuals, not only those “driving” the correlation between MA and mathematics performance and who have been the attention of researchers.

### How to apply it to classroom practice and educational policy?

From our own experience, we know that mathematics teachers are well aware that some of their students are anxious about mathematics. At the same time, we see that teachers really want to address this problem and learn from the researchers. Thus, we believe that one of the biggest challenges of MA research is bringing the knowledge accumulated in past decades to the classroom practice in a scalable way. This should not be limited to “raising awareness” and similar initiatives, which although often quite helpful do not offer immediate solutions. At the same time, it is important to avoid simplistic explanations and solutions, which sometimes appear in the public discourse (e.g., that MA is predominantly caused by a single factor). Developing proper resources that can be used by teachers is one of the biggest challenges to the field. On top of raising awareness of the teachers and education practitioners, proper screening tools need to be administered to children, which are easy to use, valid, and reliable. This would allow teachers to identify pupils who are in the process of developing MA before the anxiety affects their well‐being significantly and/or a vicious cycle of lowered mathematical performance and increased MA develops.

This, despite being challenging, should be relatively simple to implement. The difficulty is in finding ways to develop scalable and cost‐effective intervention programs, especially those targeted at emotional regulation and grounding, before approaching mathematics problems. Psychotherapy‐inspired interventions proved effective for alleviating MA.^9^ Unfortunately, such interventions typically require one‐to‐one contact with a highly trained professional, thus they incur considerable costs and are not easily implemented. Several brief interventions at school aimed at alleviating MA have been tested (e.g., see Refs. 61 and 62), though the effects of such interventions have not always been replicated.^63^ It seems reasonable to think of something between individual psychotherapy‐based interventions and brief one‐time interventions (see Refs. 61 and 62). Such programs would ideally be administered by the teacher/educational practitioner: preferably within integrated mathematics curricula that also includes focus on prevention and remediation of MA. Developing such programs should be guided by the conclusions of studies that identify those likely to benefit from MA interventions (see section above); some important considerations for development of such interventions can be found in Ref. 65. Also, game‐based interventions might be an interesting avenue of investigation.^65^ The development of such programs would certainly benefit from a constant exchange of information between researchers and practitioners. Such collaborations should be fostered both at the local and global levels so that researchers have access to “reality checks” on whether solutions they propose are feasible and applicable in a wider range of contexts.

Moreover, initiatives should not be limited to children. Early years education teachers often demonstrate relatively high MA,^66^, ^67^, ^68^, ^69^ which can lead to increased MA in their pupils.^70^ MA intervention programs, therefore, should be developed for such teachers. Moreover, intervention programs should be available to parents, as their attitudes and behaviors toward mathematics, as well as their own MA, may negatively affect childrens’ learning experiences.^71^, ^72^

### Outside educational settings

Most MA studies have been conducted in educational settings (schools, colleges, and universities). Even those that do not test current pupils/students have usually involved individuals, who were linked to some educational settings: elementary school teachers,^66^, ^70^ or parents of elementary school children (e.g., see Refs. 72, 73–74). Studies investigating adults outside the educational system are relatively scarce (with the notable exception of Hart and Ganley^51^).

On the other hand, several models of MA explicitly consider that MA can manifest itself in daily life situations (e.g., calculating change or a tip^75^, ^76^, ^77^). At the same time, we are aware of serious economic costs of low numeracy both at the individual and global levels.^78^, ^79^ For this reason, it would be worthwhile to focus on individuals who have already left education to see their MA level, its consequences, and potential means for remediation. The latter have not, to the best of our knowledge, been investigated in these groups. One of the very few studies investigating such a population^51^ has in general replicated findings from studies conducted within educational settings. Nevertheless, we believe that still a lot needs to be done to better understand MA outside academic context and possibilities for addressing it.

### Links to well‐being

Most studies looking at the consequences of MA focused on its links to mathematics performance, vocation choice, and so on. Relatively less attention was given to consequences on individual well‐being and mental health. We know that anxiety correlates negatively with well‐being.^80^ It would be worth investigating similar long‐term costs of MA, including mental health and quality of life.^81^, ^82^, ^83^ We also do not know whether in individuals who decide on pursuing mathematics‐related careers, despite having elevated MA, such a career happens at the expense of quality of life and well‐being. Chronic stress can be a serious risk factor for burnout and potentially other mental and physical health conditions,^84^ and withdrawal from specific careers (which may be more prevalent among women).^85^ Moreover, even for people who do not pursue mathematics‐related careers, there is an increasing need in the modern world to take responsibility for one's self‐budgeting, for example, in online banking and the need to make decisions about the details of pensions. Such demands may increase the stress of MA even in those who can cope cognitively with them, as well as lead to financial problems and social exclusion, and effects on well‐being for those who cannot. Another group that may experience MA impact on well‐being are individuals who decide not to pursue their passion for STEM because it requires math. Given such issues, we strongly believe that focusing research on the links of MA to well‐being would bring several theoretical and practical benefits.

**BOX 2. Pending questions in MA research**

1. How should MA be understood? Do we look at it from a clinical‐like perspective or treat it as a differential variable?
2. What is the empirical support for the validity of the MA as a construct: (a) convergence between self‐descriptive, implicit, and physiological MA measures; (b) relations between mathematics testing (evaluation) anxiety and test anxiety; (c) relations between MA and statistics anxiety.
3. Can focusing on the state–trait discrepancy in MA foster theoretical understanding of MA and intervention planning?
4. What is the prevalence of MA, and which external criteria should be used to avoid estimating the prevalence based purely on statistical criteria?
5. What can be learned by looking at individuals who perform high in mathematics and score high in MA, and from those whose MA is low despite also having low mathematics performance? Could such discrepancies be influenced by cultural factors, as well as by individual differences and local environmental factors?
6. How might MA, and its relationship to mathematical performance, interact with intrinsic and extrinsic motivation for mathematics?
7. How can MA research be applied in classroom practice and educational policy; in particular, how can scalable interventions targeting MA be prepared?
8. What happens to MA outside educational settings; what are its consequences; and how can they be alleviated?
9. What are the consequences of MA beyond those on mathematics performance, especially whether and how does it impact individuals’ well‐being?
10. When and how does MA begin? To what extent can it be seen in the early school years and even preschool years? Could very early intervention prevent it from developing?

## Conclusions

Over the past several years, there have been many studies of MA. Recent review papers, meta‐analyses, and books provide many insights and information that have added to the current knowledge about MA. Our aim in this perspective was to reflect not only on what is known but also on what MA researchers (should) want to know (Box 2). The issues and questions span from clarifying definitions and theories and to bringing new theories into practice. When pointing out deficiencies and lacunae, we do not intend to undermine the great amount and quality of MA research that has been done. On the contrary, we are optimistic that any lacunae will soon be filled with even more solid theoretical and empirical work, and eagerly wait to witness these developments.

## Author contributions

K.C. wrote an initial draft. It was critically reviewed, complemented, and discussed by F.H.S., K.K., and A.D. All authors approved the final version of the manuscript.

## Competing interests

The authors declare on competing interests.

### Peer review

The peer review history for this article is available at: https://publons.com/publon/10.1111/nyas.14770

## Footnotes

a This is only a tentative proposal, and specific thresholds for each of criterion are yet to be established.
